# Prevalence and distribution of high-risk human papillomavirus genotypes among women living with HIV in Nigeria

**DOI:** 10.1371/journal.pone.0360438

**Published:** 2026-11-10

**Authors:** Ijeoma M. Ifeorah, Jonadab Chinwendu, Stanley C. Eneh, Babayemi O. Olakunde, Uduak Akpan, Nicholas A. Awolola, Uwem E. George, Temitope O. C. Faleye, Popoola Bolutife, Chukwuma E. Omoruyi, Chinonso C. Iyi, Carey Tishiya, Emo A. Ivo, Adekunbiola A. F. Banjo, Chibuike O. Chigbu, Lifang Hou, Echezona E. Ezeanolue, Olubusuyi M. Adewumi

**Affiliations:** 1 Department of Medical Laboratory Sciences, Faculty of Health Sciences, University of Nigeria, Enugu Campus, Enugu, Nigeria; 2 Vaccine Research Center, University of Nigeria, Enugu, Nigeria; 3 IVAN Research Institute, University of Nigeria, Enugu, Nigeria; 4 Interdisciplinary Health Sciences, University of Texas at El Paso, El Paso, Texas, United States of America; 5 Department of Population and Community Health, College of Public Health, University of North Texas Health Science Center, Fort Worth, Texas, United States of America; 6 Excellence Community Education Welfare Scheme (ECEWS), Abuja, Nigeria; 7 Department of Anatomy and Molecular Pathology, Lagos University Teaching Hospital, Lagos, Nigeria; 8 Department of Biological Sciences, Faculty of Natural and Applied Sciences, Lead City University, Ibadan, Oyo State, Nigeria; 9 Center for Environmental Health Engineering, Biodesign Institute, Arizona State University, Tempe, Arizona, United States of America; 10 Department of Virology, College of Medicine, University of Ibadan, Ibadan, Oyo State, Nigeria; 11 Department of Medical Laboratory Science, Faculty of Allied Health Sciences, Federal University of Health Sciences, Ila-Orangun, Osun State, Nigeria; 12 Department of Epidemiology, University of Chicago, Chicago, Illinois, United States of America; 13 Ministry of Defense Health Implementation Program, Abuja, Nigeria; 14 Department of Preventive Medicine, Feinberg School of Medicine, Northwestern University, Chicago, Illinois, United States of America; 15 Infectious Disease Institute, College of Medicine, University of Ibadan, Ibadan, Oyo State, Nigeria; IAVI, UNITED STATES OF AMERICA

## Abstract

Women living with HIV (WLHIV) are at significantly higher risk of persistent high-risk human papillomavirus (hrHPV), and consequently, cervical cancer. However, large scale data on the epidemiology of hrHPV genotypes among WLHIV are limited in Nigeria. The study aimed to determine the prevalence and distribution of hrHPV among WLHIV in Nigeria. We conducted a cross-sectional study among 1327 WLHIV aged 25−60 years receiving HIV care from six selected Nigeria Implementation Science Alliance–Model Innovation and Research Centers (NISA-MIRCs) across Nigeria. Cervical swabs were obtained, and HPV DNA genotype was determined using the Hybribio Genoarray assay that detects 37 HPV genotypes including 14 high-risk HPVs. Sociodemographic and clinical data were collected using interviewer-administered structured questionnaire. Factors independently associated with hrHPV infection were identified using multivariable logistic regression. Statistical analysis was performed using R statistical software (version 4.4.1) with significance set at p < 0.05. Among the 1105 participants with analyzable samples, the overall HPV and hrHPV prevalence were 30.9% and 22.1%, respectively, with regional differences. Non-HPV 16 and 18 accounted for nearly two-thirds (67.6%) of hrHPV infections. The top five most often detected hrHPVs were HPV-58 (14.2%), HPV-16 (12.7%), HPV-52 (12.4%), HPV-31 (10.6%), and HPV-18 (10.3%). In a multivariate analysis, ART usage for one year or less was associated with hrHPV infection (aOR = 8.2, 95% CI 4.3–16.0). This multi-site study showed a significant and genotype-diverse burden of hrHPV among HIV-positive women in Nigeria, with most infections caused by non-HPV16/18 variants. These findings emphasize the need for increased integration of cervical cancer screening within HIV care and support the call for HPV vaccines with broader genotype coverage beyond HPV 16 and 18 to address the prevailing genotype distribution in this population.

## Introduction

Cervical cancer (CxCa), caused primarily by persistent high-risk human papillomavirus (hrHPV) infection, is a major cause of cancer-related mortality among women in sub-Saharan Africa (SSA) [1]. Women living with HIV (WLHIV) face a higher risk for acquiring and sustaining persistent hrHPV infection, making them six times more likely to have CxCa and twice the mortality risk compared to their HIV negative counterparts [2,3]. Nigeria bears one of the largest HIV burden globally with an estimated 2 million people living with HIV and women disproportionately affected [4]. Also, CxCa is the second leading cause of cancer-related deaths among women in the country [5]. Consequently, the implementation of effective CxCa prevention and control strategies, particularly those tailored to the unique needs of WLHIV, is crucial for Nigeria and SSA.

Human Papillomaviruses (HPV) are non-enveloped, double-stranded DNA viruses that infect both keratinized and non-keratinized epithelial surfaces [6]. Based on the potential to cause cancer, HPVs are broadly classified into two main groups: low-risk (lr) and high-risk (hr) HPV. Among the many identified HPV types, the International Agency for Research on Cancer (IARC) classified twelve (HPV 16, 18, 31, 33, 35, 39, 45, 51, 52, 56, 58, 59) as Group 1 carcinogens, HPV 68 as Group 2A (Probable high-risk), HPV 66, 53, 26, 82 group 2B (Probable high-risk) and many others including HPV 6 and 11 as class 3 (not classifiable as carcinogenic) [5,7]. Globally, HPV types 16 and 18 account for about 76% of CxCa [8]. While infection with hrHPV is necessary, it is not sufficient on its own to cause CxCa. Other risk factors such as coinfection with HIV and other STIs, smoking, prolonged use of oral contraceptives, socio-economic status, etc, play critical roles [2].

Approaches to CxCa control include vaccination, screening, and treatment of precancerous lesions [9]. Consequently, type-specific HPV vaccines, including bivalent, tetravalent, and nonavalent, have been introduced and are recommended for adolescents and young women aged 9–26 years in many countries [10–12]. In Nigeria, the tetravalent HPV vaccine has recently been rolled out for the vaccination of eligible adolescent girls across all the six-geopolitical zones [13]. However, the prevalence and genotype distribution of hrHPV vary among regions and populations [14–19]. Also, currently existing HPV vaccines do not protect against all identified hrHPV types. Consequently, generating reliable and population-specific data on hrHPV distribution in WLHIV is crucial to evaluate, improve, and inform effective CxCa control strategies for this population in Nigeria.

In Nigeria, comprehensive data on hrHPV distribution among WLHIV is limited. A systematic review and meta-analysis of studies in Nigeria by Ezechi *et al* [18] reported 70% hrHPV prevalence in the WLHIV subgroup from southwestern and Northcentral Nigeria, with no representation from the other four regions. Similarly, a recent study [20] covering four states in Nigeria reported an overall prevalence of 15.1%, reflecting wide variation in existing data. With the renewed interest in CxCa control in Nigeria, the need for data on hrHPV in WLHIV cannot be overemphasized. Thus, this large-scale multi-center study aimed to map the distribution of hrHPV among WLHIV across Nigeria to provide robust multi-centre data to inform and/or optimize CxCa control strategies and interventions in Nigeria.

## Methods

### Study design and setting

This was a cross-sectional study conducted in six healthcare facilities across Nigeria’s six geopolitical zones. The six study sites were selected from the Nigeria Implementation Science Alliance–Model Innovation and Research Centers (NISA-MIRCs). The NISA-MIRCs comprise 21 high-volume clinical sites that collectively provide care for more than 60,000 WLHIV nationwide [21] and have been designated for clinical and implementation research.

Six out of the twelve NISA-MIRCs participating in the Accelerating Cervical Cancer Elimination through the Integration of Screen-and-treat Services (ACCESS) study, an implementation trial evaluating the implementation strategies to integrate CC screening into the HIV program [22], were purposefully selected for this study (Fig 1).

**Fig 1:**
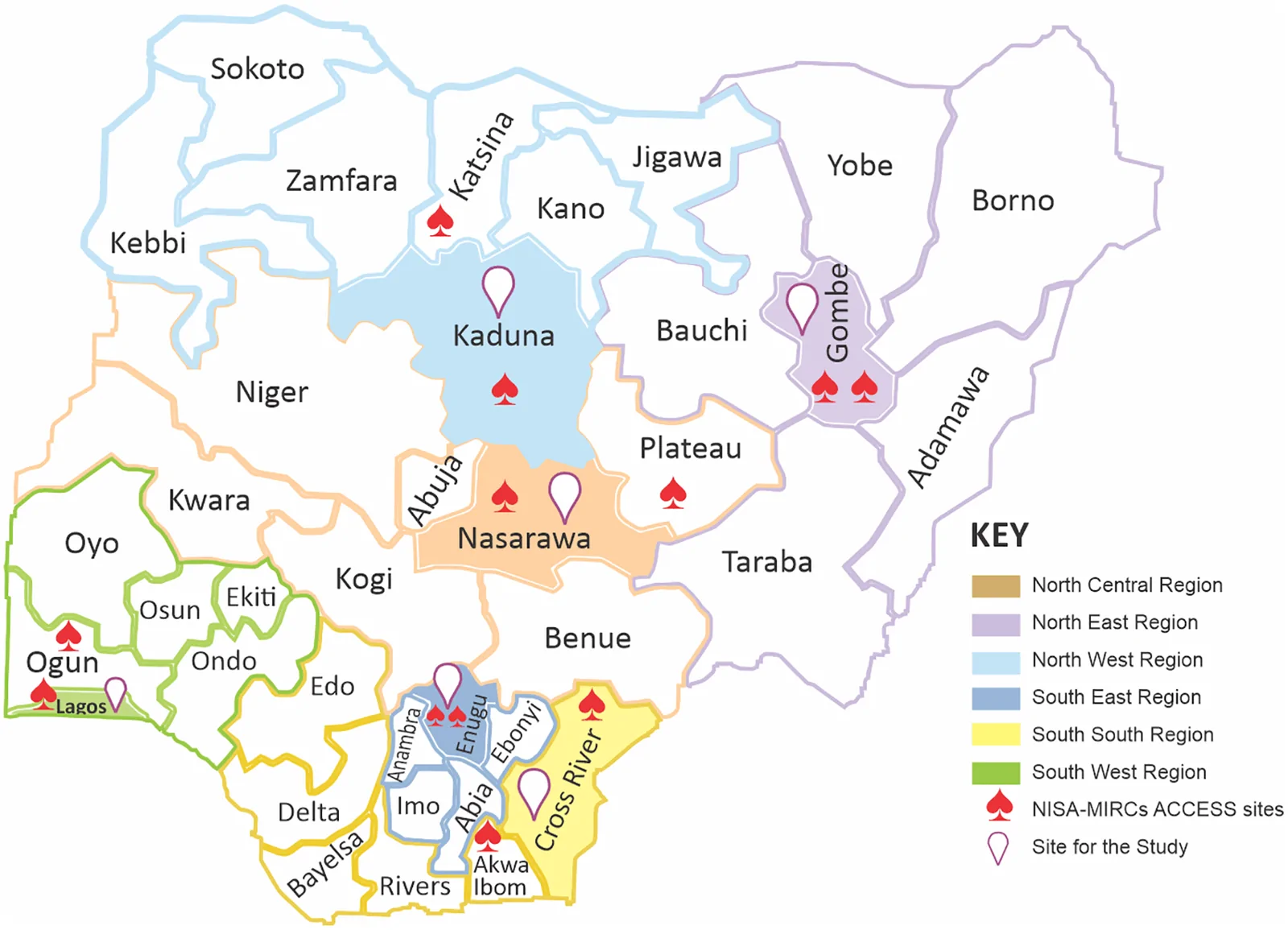
Map of Nigeria indicating the six selected study sites.

### Sample size determination and study population

Based on a high-risk HPV (hrHPV) prevalence of 19.6% among WLHIV in Lagos, Nigeria [17], a 90% confidence level, and a 5% margin of error, a sample size of 1,320 women (220 per site) across the six study sites was estimated. The sample size was calculated using Epi Info™ version 7. Between January 2025-June 2025 by convenient sampling the study recruited 1327 consenting WLHIV, aged 25–60 years, who were receiving antiretroviral care across the six study sites with relatively even distribution: Northcentral (Nasarawa; n = 225,16.9%), Northeast (Gombe; n = 220, 16.6%), Northwest (Kaduna; n = 221,16.7%), Southeast (Enugu; n = 220, 16.6%), South-south (Cross River; n = 222,16.7%) and Southwest (Lagos; n = 219, 16.5%). Women who were: (i) clinically unstable, (ii) pregnant, (iii) had a prior history of hysterectomy, or (iv) without any self-documented history of sexual intercourse were excluded from the study.

### Survey instrument and data collection

Data was collected using a pilot-tested, interviewer-administered questionnaire. The 18-item instrument was divided into two sections to collect data relating to sociodemographic characteristics and cervical cancer-associated risk factors. Data were entered into REDCap™ electronic data capture tools, with submission-lock features to prevent post-submission edits. The instrument was piloted at a non-participating NISA-MIRC site for clarity and feasibility. Trained research coordinators administered the final questionnaire across the study sites.

After questionnaire administration, a trained health nurse performed gynecological examinations and collected cervical specimens for HPV DNA using cervical brush which was placed into a well labelled 3 ml MicroTest M4RT (Fisher Scientific, USA) Transport Medium for viruses and chlamydia. At the end of recruitment MicroTest M4RT tubes containing the cytological brush were stored at −20 degrees Celsius at the collection sites until shipment to the Molecular Biology Research Laboratory, Lagos University Teaching Hospital, where HPV DNA testing was done.

### HPV genotyping

Automated HPV DNA extraction and Genotyping were done using the HybriBio (HybriBio, Hong Kong) HPV GenoArray assay, which employed an L1 consensus primer-based PCR followed by reverse line dot blot hybridization. The GenoArray detects 37 HPV genotypes (including fourteen high-risk types (types 16, 18, 31, 33,35, 39, 45, 51, 52, 56, 58, 59, 66, 68) and twenty-three others. All procedures were performed according to the manufacturer’s instructions. Briefly, PCR was performed in a Perkin-Elmer GeneAmp PCR system 9700 apparatus (Applied Biosystems) using a final reaction volume of 25 μl containing 5 μl of DNA template, 19.25 μl of master mix, and 0.75 μl of DNA *Taq* polymerase. The amplification protocol consisted of initial denaturation at 95°C for 9 minutes and 40 cycles of 20 seconds of denaturation at 95°C, 30 seconds of annealing at 55°C, and 30 seconds of elongation at 72°C, followed by a final extension for 5 minutes at 72°C.

For detection and result interpretation, the resulting PCR products were denatured at 95°C for 5 minutes and subjected to genotype-specific reverse dot blot hybridization using the GenoArray membrane-based platform. The assay utilized a flow-through hybridization technique by actively directing the targeting molecules toward the immobilized probes within the membrane fibers, with the complementary molecules being retained by the formation of duplexes. After a stringent wash, the hybrids were detected by the addition of the streptavidin-horseradish peroxidase conjugate, which binds to the biotinylated PCR products, and a substrate (nitroblue tetrazolium-5-bromo-4-chloro-3-indolylphosphate) to generate a purple precipitate at the probe dot. The results were interpreted by direct visualization.

### Quality control

The positive control provided with the kit and the two negative controls (HPV-negative C33-A cells and PCR water) were included in each set of PCRs to assess the performance of the test. After hybridization, the presence of a positive result for both the “internal control” and the “biotin” dots within the membrane indicated that the isolated DNA was of good quality, the enzyme conjugate was valid, and the hybridization process was proper.

### Statistical analysis

Data was analyzed using R statistical software (version 4.4.1). Descriptive statistics were used to summarize participants’ sociodemographic, clinical, sexual, and reproductive characteristics. Continuous variables were summarized using medians and interquartile ranges due to non‑normal distributions, while categorical variables were summarized using frequencies and percentages.

Overall, HPV and hrHPV prevalence were calculated as the proportion of participants with valid HPV test results who tested positive. Prevalence estimates were further stratified by study region and participant characteristics. Differences in HPV prevalence across categorical variables were assessed using Pearson’s chi‑squared test, while Fisher’s exact test was applied where expected cell counts were small. The distribution of hrHPV genotypes and the number of concurrent hrHPV infections per individual (single versus multiple hrHPV infections) were summarized descriptively by region and presented as figures and charts.

To identify independent predictors of hrHPV infection, multivariable logistic regression was performed, with hrHPV status (positive vs. negative) modeled as the binary outcome. Variables considered for inclusion in the multivariable model were selected based on biological plausibility, prior literature, and bivariate analyses. The final model included duration of ART usage, age, age at first sexual intercourse, number of pregnancies, number of lifetime sexual partners, educational attainment, history of other sexually transmitted infections, history of cervical cancer screening and study site. Variables with very low cell counts (including contraceptive use category, occupation, and history of chronic disease) were excluded to ensure model stability.

Data completeness varied across the variables included in the multivariable model, ranging from under 1% to about 14% (educational attainment). The main analysis included only participants with complete data on all model variables (n = 783 of 1105). To check whether this affected our results, we repeated the analysis using multiple imputations. Both approaches gave consistent results in direction and statistical significance, supporting the reliability of our findings (S1 Table).

Also, population attributable fractions (PAFs) were estimated for selected exposures identified in the multivariable analysis to quantify the proportion of hrHPV infections attributable to these risk factors at the population level. PAFs and corresponding 95% confidence intervals were derived using bootstrap resampling (1,000 iterations). All statistical tests were two‑sided, and p-values <0.05 were considered statistically significant.

### Ethics

Ethical approval was obtained from the National Health Research Ethics Board with approval number NHREC/01/01/2007-25/05/2024, and electronically signed informed consent was obtained from each participant before recruitment. All hrHPV positive participants were triaged and referred to the team’s gynecologist for follow-up and management.

## Results

### Demographic and clinical characteristics of the study population

HPV test results for a significant number of samples from South-South region were invalid, possibly due to poor sample storage and handling issues. During quality control review it was also observed that available results from this site showed high degree of similarity in genotype profiles across samples raising concern about their reliability. Consequently, downstream data analysis and result presentation included only 1105 (83.3%) participants (median age 41 years (IQR 36–47) from the other five sites (Fig 2).

**Fig 2:**
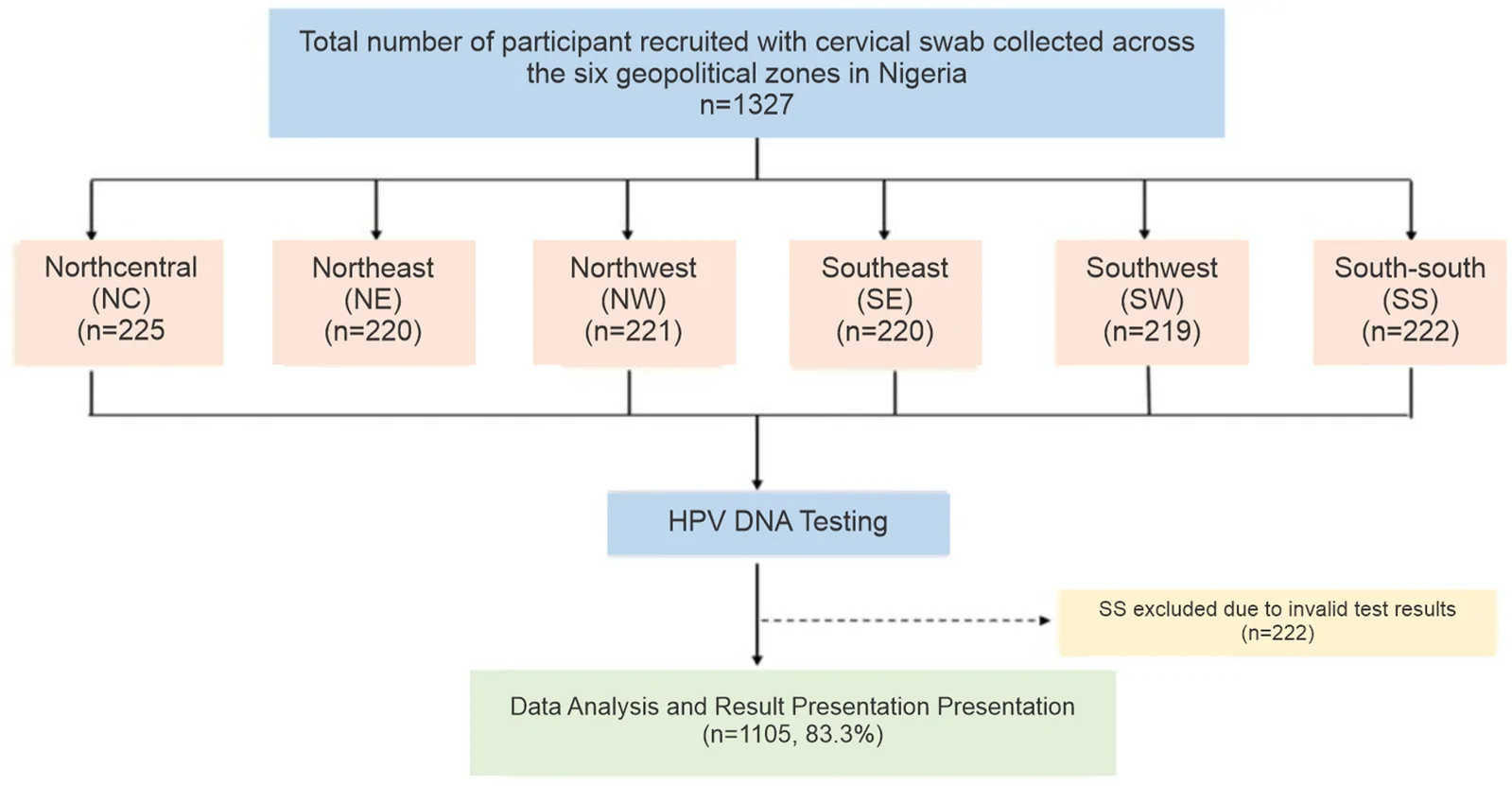
Flowchart of recruited participants, HPV DNA Testing and inclusion in the final analysis. 1327 cervical swabs were collected from all participants and tested for HPV DNA using Hybribio HPV GeneArray Assay. HPV DNA test result for participants from South-south Nigeria were invalid and were excluded from the final analysis and result presentation.

Most participants (65.9%) attained at least secondary education, while 20.2% had primary education and 13.9% had no formal education. Regarding marital status, 58.6% reported being married, while 11.3% were never married. The remaining others were either widowed (20.3%) or divorced/separated (9.8%). The majority (80.6%) of the women were in unskilled/vocational labour, while 14.3% and 5.1% were either in skilled employment or unemployed, respectively (Table 1). The median age at sexual debut was 20 years (IQR 18–21) with nearly two-thirds (67.7%) of the women reporting first sexual intercourse between 18 and 24 years, while 21.3% initiated sexual intercourse before 18years. Only 104 (10.5%) reported early age (<18 years) at first pregnancy. Overall, 824 (74.6%) of the participants reported no previous use of contraceptives. Additionally, 53.7% had experienced four or more pregnancies. The median for the number of reported lifetime sexual partners was 2 (IQR 1,3) (Table 1). Most (927; 84.0%) of the women had been on ART for five years or more, while only 72 (6.5%) had been on ART for a year or less. Among those with reported histories of comorbidities and other sexually transmitted infections (STIs), 1010 (91.4%) and 891 (80.6%) reported no history of chronic disease or prior other STIs, respectively. Prior cervical cancer screening coverage was moderate, with 656 (59.4%) participants reporting having been screened in the past (Table 1).

**Table 1:** Sociodemographic and clinical characteristics of the study population.

Characteristic	Category	Total (%)	NC (NAS) (%)	NE (GMB) (%)	NW (KAD) (%)	SE (ENU) (%)	SW(LAG) (%)
	No Recruited*	1,105	225 (20.4)	220 (19.9)	221(20.0)	220 (19.9)	219 (19.8)
Age at last birthday (years)	25-40	525 (47.5)	123 (11.1)	133 (12.0)	76 (6.9)	97 (8.8)	96 (8.7)
	> 40	580 (52.5)	102 (9.2)	87 (7.9)	145 (13.1)	123 (11.1)	123 (11.1)
	Median (Q1, Q3)	41 (37, 47)	40 (35, 47)	40 (35, 46)	43 (39, 48)	42 (37, 47)	42 (36, 49)
Highest formal education attained	Post-secondary	267 (24.2)	60 (5.4)	32 (2.9)	45 (4.1)	82 (7.4)	48 (4.3)
	Secondary	461 (41.7)	55 (5.0)	68 (6.2)	113 (10.2)	108 (9.8)	117 (10.6)
	Primary	223 (20.2)	43 (3.9)	51 (4.6)	48 (4.3)	30 (2.7)	51 (4.6)
	None	154 (13.9)	67 (6.1)	69 (6.2)	15 (1.4)	0 (0.0)	3 (0.3)
Marital status	Married	648 (58.6)	128 (11.6)	145 (13.1)	104 (9.4)	145 (13.1)	126 (11.4)
	Never Married	125 (11.3)	18 (1.6)	13 (1.2)	32 (2.9)	36 (3.3)	26 (2.4)
	Separated/Divorced	108 (9.8)	19 (1.7)	25 (2.3)	26 (2.4)	7 (0.6)	31 (2.8)
	Widowed	224 (20.3)	60 (5.4)	37 (3.3)	59 (5.3)	32 (2.9)	36 (3.3)
Occupation	Skilled	158 (14.3)	47 (4.3)	15 (1.4)	15 (1.4)	47 (4.3)	34 (3.1)
	Unskilled	891 (80.6)	163 (14.8)	181 (16.4)	203 (18.4)	162 (14.7)	182 (16.5)
	Unemployed	56 (5.1)	15 (1.4)	24 (2.2)	3 (0.3)	11 (1.0)	3 (0.3)
Age at coitarche (years)	< 18	235 (21.3)	59 (5.3)	104 (9.4)	9 (0.8)	19 (1.7)	44 (4.0)
	18-24	748 (67.7)	147 (13.3)	98 (8.9)	209 (18.9)	148 (13.4)	146 (13.2)
	>=25	122 (11.0)	19 (1.7)	18 (1.6)	3 (0.3)	53 (4.8)	29 (2.6)
	Median (Q1, Q3)	20.0 (18.0, 21.0)	19.0 (17.0, 21.0)	18.0 (16.0, 20.0)	20.0 (19.0, 21.0)	20.0 (19.0, 24.0)	19.0 (18.0, 22.0)
Age at first pregnancy (years)	<18	104 (10.5)	28 (2.8)	56 (5.6)	4 (0.4)	9 (0.9)	7 (0.7)
	18-30	839 (84.6)	173 (17.4)	144 (14.5)	194 (19.6)	141 (14.2)	187 (18.9)
	>=30	49 (4.9)	4 (0.4)	5 (0.5)	2 (0.2)	21 (2.1)	17 (1.7)
	Median (Q1, Q3)	22.0 (19.0, 25.0)	21.0 (19.0, 23.0)	19.0 (17.0, 22.0)	23.0 (22.0, 24.0)	24.0 (20.0, 28.0)	23.0 (20.0, 26.0)
Contraceptives use	Yes	281 (25.4)	66 (6.0)	34 (3.1)	82 (7.4)	23 (2.1)	76 (6.9)
	No	824 (74.6)	159 (14.4)	186 (16.8)	139 (12.6)	197 (17.8)	143 (12.9)
Number of pregnancies	0	113 (10.2)	20 (1.8)	15 (1.4)	21 (1.9)	49 (4.4)	8 (0.7)
	1-3	450 (45.4)	71 (7.2)	75 (7.6)	102 (10.3)	98 (9.9)	104 (10.5)
	>=4	542 (54.6)	134 (13.5)	130 (13.1)	98 (9.9)	73 (7.4)	107 (10.8)
	Median (Q1, Q3)	4.00 (2.00, 5.00)	5.00 (3.00, 6.00)	4.00 (3.00, 6.00)	3.00 (2.00, 5.00)	3.00 (2.00, 5.00)	4.00 (2.00, 5.00)
Number of lifetime sexual relationships	1	284 (25.7)	43 (3.9)	97 (8.8)	102 (9.2)	17 (1.5)	25 (2.3)
	2-4	669 (60.5)	149 (13.5)	107 (9.7)	119 (10.8)	175 (15.8)	119 (10.8)
	>=5	152 (13.8)	33 (3.0)	16 (1.4)	0 (0.0)	28 (2.5)	75 (6.8)
	Median (Q1, Q3)	2.00 (1.00, 3.00)	2.00 (2.00, 3.00)	2.00 (1.00, 3.00)	2.00 (1.00, 2.00)	3.00 (2.00, 3.00)	3.00 (2.00, 5.00)
ART duration (years)	<= 1	72 (6.5)	1 (0.1)	8 (0.7)	6 (0.5)	25 (2.3)	32 (2.9)
	2-4	105 (9.5)	7 (0.6)	12 (1.1)	18 (1.6)	35 (3.2)	33 (3.0)
	>=5	927 (84.0)	217 (19.7)	200 (18.1)	197 (17.8)	159 (14.4)	154 (13.9)
	Median (Q1, Q3)	10.0 (6.0, 15.0)	13.0 (10.0, 16.0)	11.0 (8.0, 15.0)	11.0 (8.0, 15.0)	10.0 (4.0, 15.0)	7.0 (3.0, 10.0)
History of other sexually Transmitted Infections	Yes	214 (19.4)	124 (11.2)	18 (1.6)	0 (0.0)	24 (2.2)	48 (4.3)
	No	891 (80.6)	101 (9.1)	202 (18.3)	221 (20.0)	196 (17.7)	171 (15.5)
History of chronic disease	Yes	95 (8.6)	35 (3.2)	25 (2.3)	0 (0.0)	5 (0.5)	30 (2.7)
	No	1,010 (91.4)	190 (17.2)	195 (17.6)	221 (20.0)	215 (19.5)	189 (17.1)
Ever screened for cervical cancer	Yes	656 (59.4)	170 (15.4)	120 (10.9)	140 (12.7)	118 (10.7)	108 (9.8)
	No	449 (40.6)	55 (5.0)	100 (9.0)	81 (7.3)	102 (9.2)	111 (10.0)

NC = North-Central, NE = North-East, NW = North-West, SE = South-East, SW = South-West, NAS = Nasarawa, GMB = Gombe, KAD = Kaduna, ENU = Enugu, LAG = Lagos, ART = Anti-retroviral therapy, No Recruited* = Participants with analyzable sample

### Overall HPV prevalence

Of the 1,105 participants, 341 WLHIV tested positive for any of the 37 HPV types detectable by the assay, resulting in an overall HPV prevalence of 30.9% (Table 2). HPV prevalence varied significantly (p = 0.005) across study sites, with the lowest prevalence (23.1%) in NC (NAS) and the highest (36.5%) in SW (LAG).

**Table 2:** HPV Prevalence in the study population.

Variable	Category	Tested	Positive	HPV prevalence (%)	p-value
HPV Prevalence	Overall	1,105	341	30.9	
Region	NC (NAS)	225	52	23.1	
	NE (GMB)	220	76	34.5	
	NW (KAD)	221	57	25.8	
	SE (ENU)	220	76	34.5	
	SW(LAG)	219	80	36.5	**0.005**
Age at last birthday (years)	> 40	580	177	30.5	
	25-40	525	164	31.2	0.8
Highest formal education attained	None	154	47	30.5	
	Post-secondary	267	88	33.0	
	Primary	223	71	31.8	
	Secondary	461	135	29.3	0.8
Marital status	Married	648	196	30.2	
	Never Married	125	49	39.2	
	Separated/Divorced	108	32	29.6	
	Widowed	224	64	28.6	0.2
Occupation	Skilled	158	42	26.6	
	Unskilled	891	279	31.3	
	Unemployed	56	20	35.7	0.4
Age at first sexual intercourse (years)	< 18	235	86	36.6	
	18-24	748	220	29.4	
	>=25	122	35	28.7	0.1
Age at first pregnancy (years)	<18	104	34	32.7	
	18-30	839	249	29.7	
	>=30	49	14	28.6	0.8
Previous contraceptive use	No	824	255	30.9	
	Yes	281	86	30.6	>0.9
Number of pregnancies	>=4	542	163	30.1	
	1-3	450	134	29.8	>0.9
	0	113	44	38.9	
Number of lifetime sexual relationships	1	284	74	26.1	
	2-4	669	206	30.8	
	>=5	152	61	40.1	**0.01**
ART duration (years)	<=1	72	46	63.9	
	2-4	105	35	33.3	
	>=5	927	260	28.0	**<0.001**
History of Other Sexually Transmitted Infections	No	891	281	31.5	
	Yes	214	60	28.0	0.3
History of chronic disease	No	1,010	307	30.4	
	Yes	95	34	35.8	0.3
Ever screened for cervical cancer	No	449	152	33.9	
	Yes	656	189	28.8	0.075

NC = North-Central, NE = North-East, NW = North-West, SE = South-East, SW = South-West, NAS = Nasarawa, GMB = Gombe, KAD = Kaduna, ENU = Enugu, LAG = Lagos. ART = Anti-retroviral therapy.

HPV prevalence did not differ significantly by age group, formal educational attainment, marital status, occupation, age at sexual debut, age at first pregnancy, previous contraceptive use, cervical cancer screening history, history of other STI and chronic disease.(all p > 0.05) Participants who had been on ART for ≤1 year had the highest HPV prevalence (63.9%), compared with those who had been on ART for 2–4 years (33.3%) and ≥5 years (28.0%) (p < 0.001). Additionally, HPV prevalence increased with the number of lifetime sexual relationships with women reporting five or more partners having the highest prevalence (40.1%) (p < 0.01) (Table 2).

### High-risk HPV prevalence

Altogether, 244 study participants tested positive for at least one high-risk HPV (hrHPV) type, yielding an overall hrHPV prevalence of 22.1%. Varied hrHPV prevalence was noted across study sites, with the lowest prevalence (15.4%) in NW (KAD) and the highest (27.3%) in NE (GMB). High-risk HPVs were responsible for 71.6% of all HPV infections and were consistently high across all sites, ranging from 59.6% in NW (KAD) to 78.9% in NE (GMB). This suggests that most HPV infections in this population involve high-risk types (Table 3). HrHPV prevalence did not vary significantly with study population demographic characteristics. Also, women who initiated sexual activity at <18 years demonstrated higher hrHPV prevalence (26.0%) compared with a range between 20.7% − 23.0% in those reporting later sexual debut. HrHPV prevalence tended to be numerically higher in those reporting multiple lifetime sexual partners, particularly ≥5 partners (26.3%), compared to those who reported one lifetime sexual partner (16.9%). Markedly higher hrHPV prevalence (55.6%) was noted in participants with shorter ART duration (≤1 year), while those who had been on ART for ≥5 years had lower prevalence (19.3%) (p = 0.043). No significant difference in hrHPV prevalence was seen among WLHIV with a history of other sexually transmitted infections and other chronic disease and those who reported no such history. Overall, these findings highlight substantial regional heterogeneity and underscore the importance of sexual and HIV-related factors in hrHPV distribution among women living with HIV.

**Table 3:** Prevalence of high-risk HPV by participants characteristics and region.

Variable	Category	Tested	Positive	Hr-HPV prevalence (%)	p-value
Region	NC (NAS)	225	38	16.9	
	NE (GMB)	220	60	27.3	
	NW (KAD)	221	34	15.4	
	SE (ENU)	220	55	25.0	
	SW(LAG)	219	57	26.0	
	Total	1105	244	22.1	0.20
Proportion of hrHPV in HPV positive across the regions	NC (NAS)	52	38	73.1	
	NE (GMB)	76	60	78.9	
	NW (KAD)	57	34	59.6	
	SE (ENU)	76	55	72.3	
	SW(LAG)	80	57	71.3	
	Total	341	244	71.6	>0.9
Age at last birthday (years)	25-40	525	119	22.7	
	> 40	580	125	21.6	0.7
Highest formal education attained	None	154	32	20.8	
	Primary	223	51	22.9	
	Secondary	461	97	21.0	
	Post-secondary	267	64	24.0	>0.9
Marital status	Never Married	125	37	29.6	
	Married	648	146	22.5	
	Separated/Divorced	108	20	18.5	
	Widowed	224	41	18.3	0.2
Occupation	Skilled	158	28	17.7	
	Unskilled	891	203	22.8	
	Unemployed	56	13	23.2	0.6
Age at first sexual intercourse (years)	< 18	235	61	26.0	
	18-24	748	155	20.7	
	>=25	122	28	23.0	0.5
Age at first pregnancy (years)	<18	104	25	24.0	
	18-30	839	178	21.2	
	>=30	49	10	20.4	>0.9
Contraceptives use	Yes	281	61	21.7	
	No	824	183	22.2	0.9
Number of pregnancies	0	113	31	27.4	
	1-3	450	97	21.6	
	>=4	542	116	21.4	0.8
Number of lifetime sexual partners	1	284	48	16.9	
	2-4	669	156	23.3	
	>=5	152	40	26.3	0.11
ART duration (years)	<= 1	72	40	55.6	
	2-4	105	25	23.8	
	>=5	927	179	19.3	**0.043**
History of other Sexually Transmitted Infections	Yes	214	44	20.6	
	No	891	200	22.4	0.7
History of chronic disease	Yes	95	25	26.3	
	No	1010	219	21.7	0.8
Ever screened for cervical cancer	Yes	656	135	20.6	
	No	449	109	24.3	>0.9

NC = North-Central, NE = North-East, NW = North-West, SE = South-East, SW = South-West, NAS = Nasarawa, GMB = Gombe, KAD = Kaduna, ENU = Enugu, LAG = Lagos. ART = Anti-retroviral therapy.

In multivariable logistic regression analysis, ART duration of ≤1 year was independently associated with higher odds of hrHPV infection (aOR = 8.2, 95% CI 4.3–16.0, p < 0.001), compared with those who had been on ART for five or more years. Age, educational attainment, Age at first sexual intercourse, number of pregnancies, number of lifetime sexual partners, other STI history, cervical cancer screening history, and individual study site were not independently associated with hrHPV infection after adjustment (Table 4).

**Table 4:** Multivariate analysis: Predictors of high-risk HPV infection in the study participants.

Characteristic		Adjusted OR	95% CI	p-value
Region	SE (ENU)	Reference		
	SW (LAG)	1.0	0.6, 1.8	0.900
	NC (NAS)	0.9	0.5, 1.7	0.715
	NE (GMB)	1.6	0.9, 2.9	0.124
	NW (KAD)	0.8	0.4, 1.5	0.472
Age at last birthday (years)	> 40	Reference		
	25-40	1.0	0.7, 1.4	0.952
Highest formal education attained	None	Reference		
	Post-secondary	1.3	0.7, 2.3	0.454
	Primary	1.0	0.6, 1.8	0.947
	Secondary	0.9	0.5, 1.5	0.575
Age at first sexual intercourse (years)	< 18	Reference		
	18-24	0.8	0.5, 1.2	0.292
	>=25	0.8	0.4, 1.6	0.540
Number of pregnancies	>=4	Reference		
	1-3	1.0	0.7, 1.4	>0.905
Number of lifetime sexual partners	>=5	Reference		
	2-4	0.9	0.5, 1.5	0.634
	1	0.6	0.3, 1.1	0.110
ART duration (years)	>=5	Reference		
	2-4	1.0	0.5, 1.8	0.978
	<= 1	8.2	4.3, 16.0	**<0.001**
History of other Sexually Transmitted Infections	Yes	Reference		
	No	1.2	0.8, 2.1	0.362
Ever screened for cervical cancer	Yes	Reference		
	No	0.8	0.6, 1.2	0.298

No evidence of multicollinearity was observed: All GVIF^(1/(2*Df)) values ≈ 1.03–1.06. AIC = 903.8. Adjusted for all variables listed in the table. Removed in model: Variables having low counts (Contraceptive use category, History of chronic disease, occupation). Analysis included only participants with complete data; a sensitivity check using multiple imputation gave consistent results. GVIF = Generalized Variance Inflation Factor, Df = Degrees of freedom, AIC = Akaike Information Criterion.

Population attributable fractions (PAFs) were estimated to quantify the proportion of hrHPV infections attributable to selected exposures identified in the multivariable model. The PAF for short duration of ART use (≤1 year) (vs. ≥ 5 years) was 14.8% (95% CI: 9.9%–19.8%), indicating that approximately one in seven hrHPV infections in the population were statistically associated with shorter duration of ART use, under the assumptions of the model (Table 5).

**Table 5:** Population attributable fraction for high-risk HPV infection.

Exposure	PAF (%)*	95% CI lower limit)	95% CI upper limit)
Short duration of ART (≤1 year)	14.8	9.9	19.8

* Population attributable fractions (PAFs). Confidence interval (CI) derived using 1000 bootstrap resamples. Note: covariates used in the PAF estimation are the same variables retained in the multivariable logistic regression model.

### High-risk HPV distribution in the study population

Overall, the five most common hrHPV genotypes were HPV‑58 (14.2%), HPV‑16 (12.7%), HPV‑52 (12.4%), HPV‑31 (10.6%), and HPV‑18 (10.3%). This distribution pattern was nearly consistent across the study sites (Fig 3a-f).

**Fig 3:**
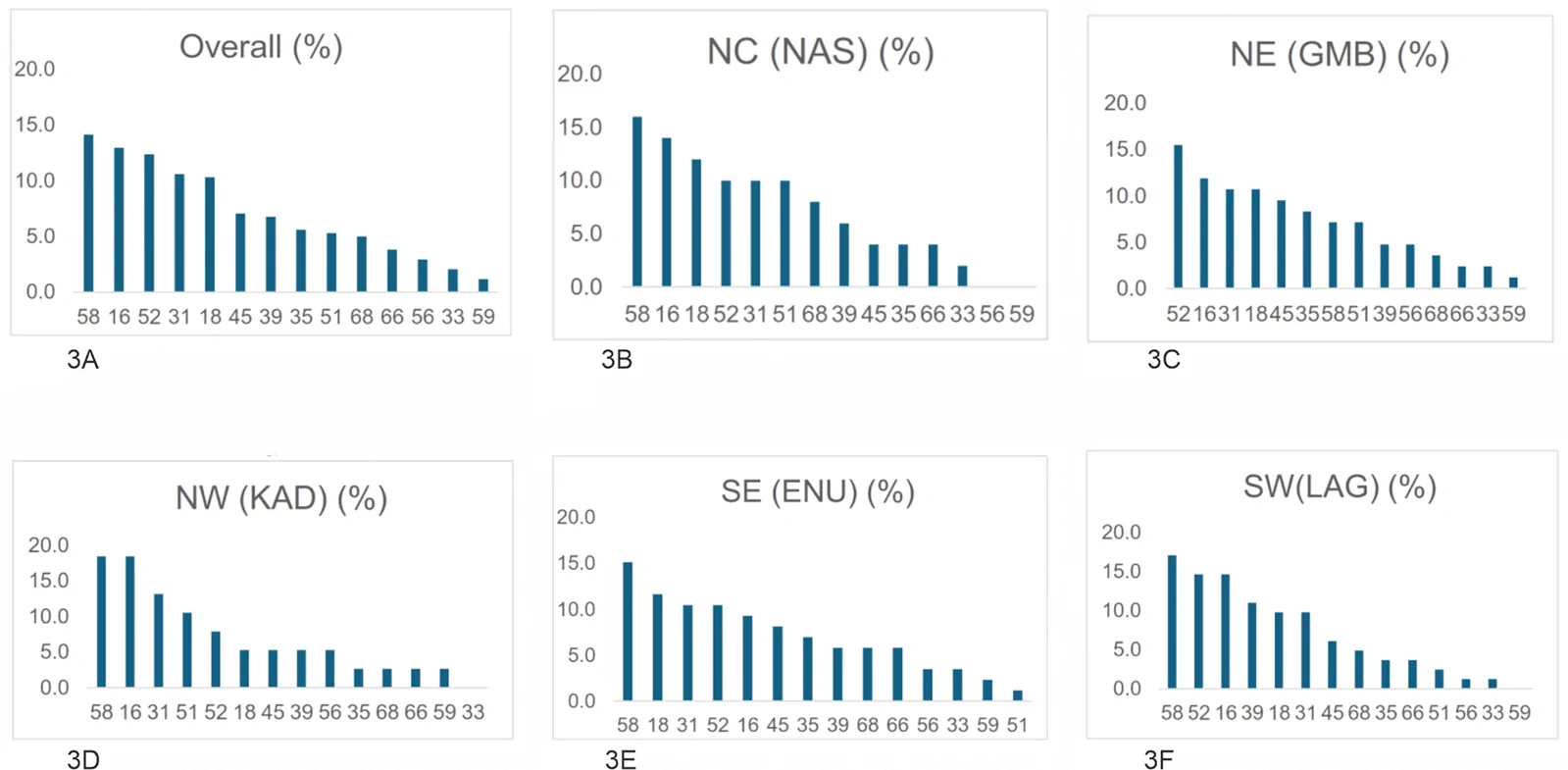
a-f. Distribution Pattern of HrHPV Detected in the study. a = Distribution pattern across all the study sites; b-e = Pattern seen in different region b = NorthCentral (NAS), c = Northeast (GMB), d = Southeast (ENU), e = Northwest (KAD), f = Southwest (LAG).

Regarding the distribution of hrHPV covered by bivalent and tetravalent HPV vaccines, 79 (32.4%) were infected with HPV types 16 and/or 18, while 165 (67.6%) harbored non-16/18 hrHPV types (Fig 4a). Thus, hrHPV genotypes not covered by the currently deployed vaccine in Nigeria accounted for nearly two-thirds of hrHPV infections in this study. Participants were mostly infected with one hrHPV type, as single infection constituted about 174 (71.3%) of the hrHPV infection, while 52 (21.3%) and 18 (7.4%) had dual hrHPV infections, and more than two hrHPV types, respectively (Fig 4b). The extent of multiple hrHPV infections varied by study sites, with SE, SW, and NE demonstrating relatively higher proportions of participants infected with two or more hrHPV types. In contrast, NC and NW were dominated by single hrHPV infections (Fig 4c).

**Fig 4:**
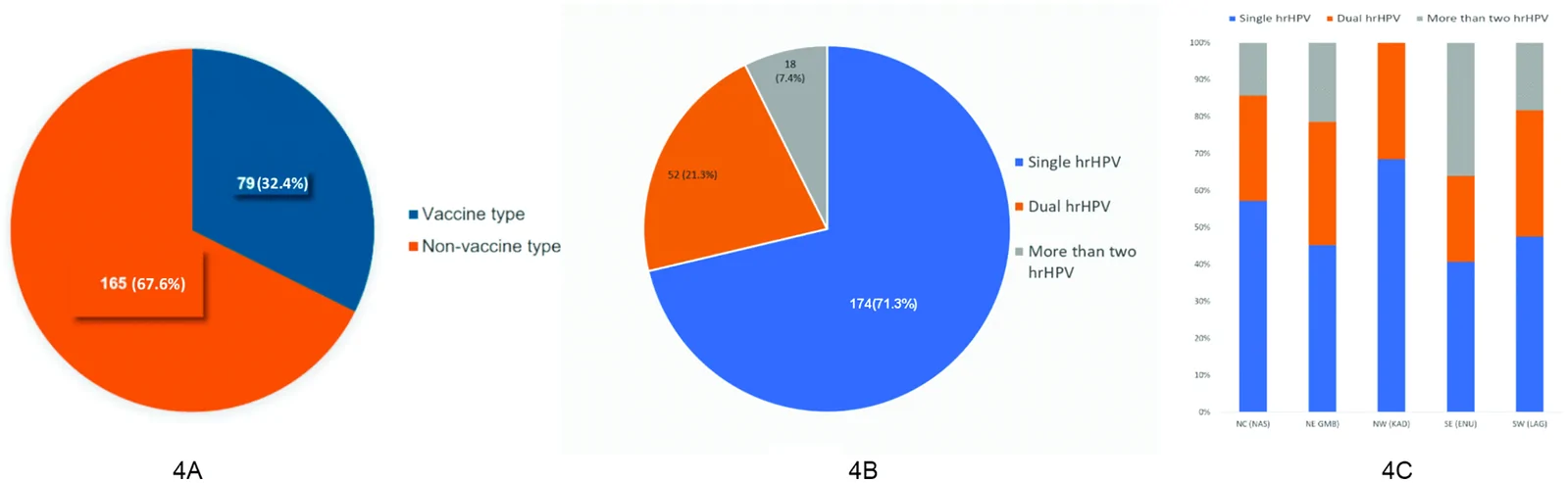
a. Proportion of study population with HPV 16/18 and Non-HPV 16/18 infection. b. Prevalence of single and multiple high risk HPV infection in the study population. c. Prevalence of single and multiple high risk HPV infection in the study population.

Regarding the proportion of individual hrHPV genotypes that occurred as single or multiple infections, about 55% of HPV 16 occurred as a single infection, while 43% of HPV 18 occurred as a single infection (Fig 5).

**Fig 5:**
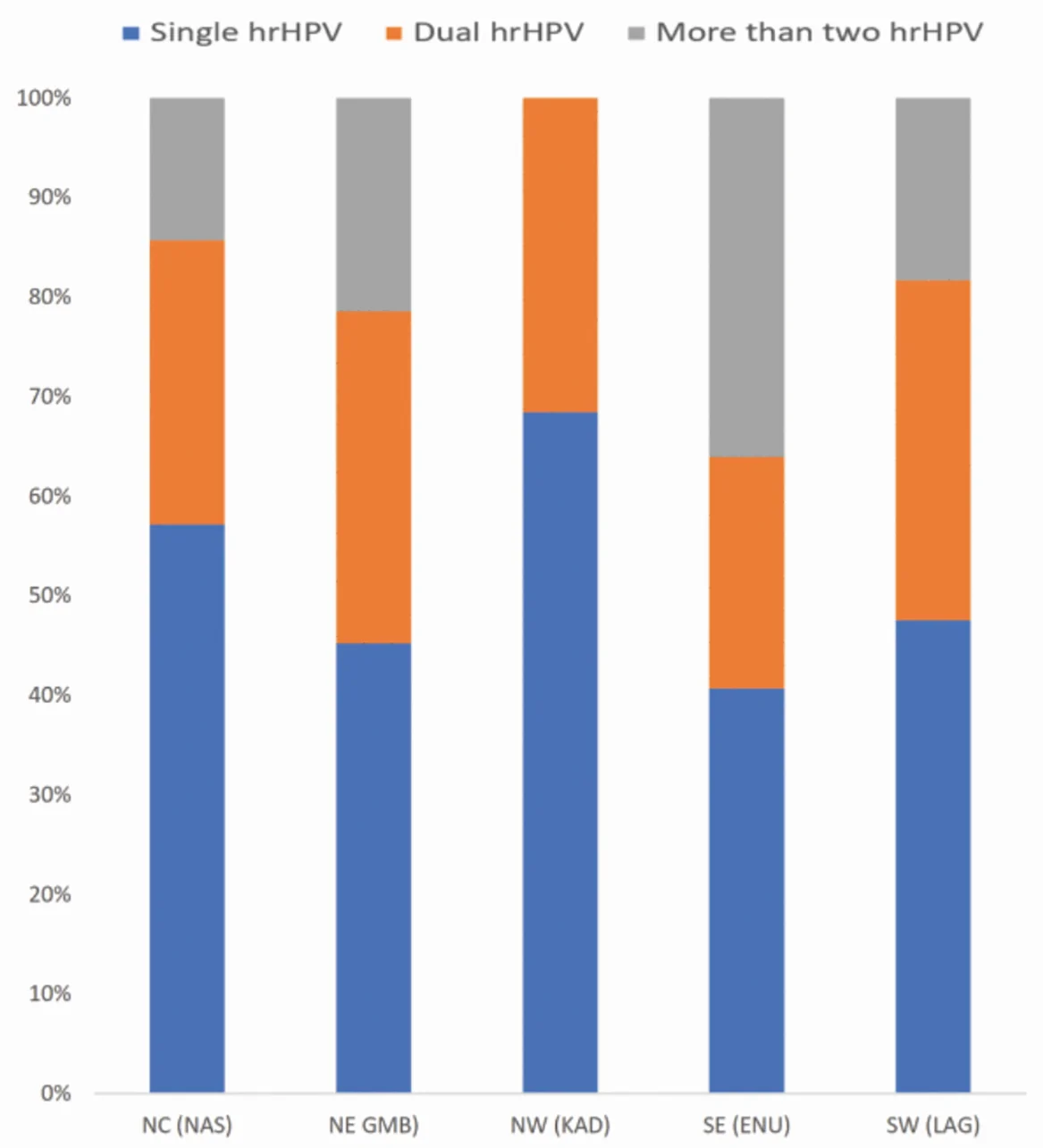
Proportion of Specific hrHPV genotypes occurring as single and multiple infections.

## Discussion

This large-scale multi-site study presents comprehensive data on hrHPV prevalence and distribution in WLHIV across Nigeria. We observed a substantial prevalence of hrHPV, which constituted about 71.6% of all HPV infections and an overall prevalence of 22.1% with some regional variations (Table 3). Interestingly, HPV 16/18 mainly targeted by the current vaccination program in Nigeria, accounted for only about one-third of all hrHPV infections, while types such as HPV-58, 52, and 31 were also frequently detected (Figs 3a and 4a). Though HPV16/18 are responsible for nearly 76% of all cervical cancers [8], evidence from many studies suggests that other hrHPV types beyond HPV 16/18 contribute significantly to cervical neoplasia and cancers [23–25]. Furthermore, shorter duration of ART use was found to be associated with HrHPV infection, with women who had been on ART for a year or less showing higher odds (aOR = 8.2, 95% CI 4.3–16.0) of hrHPV infection compared to those who had taken ART for 5 years or longer (Table 4). Importantly up to 40.6% of the study population have never had cervical cancer screening. Taken together, these findings highlight the urgent need for early cervical cancer screening following HIV diagnosis, sustained integration of HPV prevention into HIV care program and consideration for broader genotype coverage in screening and vaccination policies tailored to WLHIV.

The observed overall prevalence of 30.9% for HPV infection, with a significant regional variation (p = 0.005) indicates substantial HPV burden in the study population (Table 2). The rate of 30.9% for any HPV types obtained is comparable to previously reported rates ranging from 28%− 35% in similar cohorts from different countries in Africa, including Nigeria [26–29]. Conversely, a recent systematic review and meta-analysis [30] of studies conducted between 2019–2024 among WLHIV in SSA involving a total of 9954 participants reported a pooled prevalence of 49.9% (95% C. I = 42.43–56.38%), though with high heterogeneity reflecting significant differences between studies. The differences between the observed HPV prevalence noted in this study and the above-mentioned studies may be due to regional, population, and HPV genotyping methods variations across studies. Variation in HPV prevalence among regions and populations is documented [15]. Also, different HPV DNA detection platforms vary in sensitivity and specificity [31].

About 71.6% of all HPV infections comprised of hrHPV infection, indicating many of the HPV positive WLHIV were infected by at least one hrHPV (Table 3). The preponderance of hrHPV agrees with the expectation that WLHIV are more likely to be infected with hrHPV, which increases their risk for CxCa. Similar studies in other low- and middle-income countries have reported high proportions (51.0%− 67.2%) of hrHPV in WLHIV with HPV infection [32,33].

The overall hrHPV prevalence of 22.1%, with variations across study sites ranging between 15.4% in the Northwest (KAD) to 27.3% in the Northeast (GMB), falls within the rates of 15.1% − 28.8% reported in Nigeria and other African countries [20,34,35]. A recent large-scale study of WLHIV spanning four states in Nigeria also reported varying prevalences ranging from 13.1% in Kebbi (NE) to 18.7% in Lagos (SW) [20]. In contrast, a pooled rate as high as 71% (95% CI: 0.69, 0.73) was reported in a systematic and meta-analysis report of hrHPV in Nigerian WLHIV [18]. Similarly, in another review of studies on hrHPV infection among WLHIV in Africa [30] indicated a pooled prevalence of 49% (95% C. I = 42.43–56.38%) with the highest and lowest rates from studies from Central and Northern Africa, respectively. The lower rate of 22.1% in this study compared to the previously mentioned review may reflect differences in assay methods employed.

Regarding hrHPV distribution, HPV 16/18 accounted for only 32.4% of hrHPV infections in this study, which align with observations from another study in WLHIV from Nigeria, where non 16/18 accounted for the majority of hrHPV [20]. While HPV 16 and 18 are known to be responsible for nearly 76% of cervical cancers worldwide, other hrHPV also play significant roles. HPV 58, HPV 16, HPV 52, HPV 31, and HPV 18 were the five most frequently detected types in this study. This pattern is consistent with genotype distributions reported among WLHIV in SSA. For example, in studies from eastern Africa, hrHPV types 52, 16, and 58 were found to be the predominant genotype in WLHIV [36,37]. Similarly, in Benin and Mali, HPV58, HPV 16 and 52, and HPV 16, HPV 51 and HPV 52 were the most frequently observed, respectively [38]. This distribution pattern has implications for the HPV vaccination program in Nigeria, where the tetravalent HPV vaccine (targeting only HPV 6/11/16/18) is being widely deployed, leaving gaps for other frequently detected hrHPV types. Consistent with other reports [20,39] many of the women in our study had single hrHPV infections, suggesting that whereas women may be exposed to several hrHPV types, most infections occur as isolated genotype events rather than concurrent multi-type infections.

In this study, women who reported five or more lifetime partners had the highest HPV prevalence (any HPV types detectable by the assay employed) in our study, which may indicate higher cumulative exposure to HPV (Table 2). This supports the documented evidence of the role of sexual behaviors in HPV transmission [1,40] and reinforces the need for comprehensive sexual health education and promotion of safer sexual practices in WLHIV. Also, women who had been on ART for ≤ I year had a significantly higher HPV prevalence (any HPV) when compared with women who had a more prolonged ART duration (Table 2). Long-term antiretroviral treatment may boost immunological reconstitution, thus enhancing control of HPV infection. However, being a cross-sectional study, recent initiation of therapy may, rather than having a direct temporal effect on HPV infection, reflect ongoing high-risk sexual behavior. Similarly, in multivariable analysis, women with ART duration under ≤1 year had more than eightfold higher odds of hrHPV infection compared to those who had received treatment for ≥5 years (Table 4). Ermel *et al* [41] in a longitudinal study among WLHIV in Kenya showed that longer duration of ART use was associated with lower risk of any HPV detection. Precisely in the study, each additional year of ART use reduced the likelihood of HPV detection by 10–15%. Similarly, a cross-sectional study from an Ethiopian cohort found that ART duration of < 2 years was associated with three times the odds of hrHPV infection [35]. This finding could indicate immunological dysregulation and poor immune reconstitution during early HIV infection, which can limit HPV clearance and encourage the persistence of hrHPV strains. When ART is initiated, it takes time for the immune system to recover and regain the ability to effectively clear and control HPV infection [42,43]. Thus, the observed association between short-term ART use and hrHPV infection may reflect incomplete immune reconstitution, including the restoration of HPV-specific cell-mediated immunity. Incorporating early HPV screening into HIV care programs is important. According to the population attributable fraction, roughly 14.8% of hrHPV infections were statistically associated with shorter ART duration (Table 5), highlighting the need for enhanced surveillance in women with recent HIV diagnosis.

### Limitations

The study has some limitations. First, because of its cross-sectional nature, we could not infer a causal relationship, limiting our ability to determine whether short-term ART use directly increases hrHPV infection risk or reflects persistent high-risk behaviors. Second convenient sampling approach used may have introduced selection bias thus finding may not fully represent all WLHIV in Nigeria as clinical, behavioral and demographic characteristics which may influence HPV prevalence and distribution may differ in WLHIV who are not retained in care Third, comprehensive immunologic and treatment-related characteristics such as CD4 count, HIV viral load, and ART adherence were not included, which limits the mechanistic interpretation of the observed relationships. Finally, the exclusion of one geopolitical zone may reduce the entire national representation of our findings. Despite these limitations, the work provides valuable multi-site genotype-specific data on WLHIV in Nigeria.

## Conclusion

In summary, this large multi-site study provides genotype-specific evidence of a significant hrHPV burden among WLHIV in Nigeria with regional heterogeneity, highlights the preponderance of high-risk HPV genotypes not covered by bivalent and tetravalent vaccines, and identifies short ART duration as a key determinant of hrHPV infection. These findings provide critical insights for integrating cervical cancer prevention into HIV care and supports consideration for the use of nonavalent HPV vaccine which covers most of the hrHPV types detected in this study.

## Supporting information

S1 Table Comparison of complete-case and multiple-imputation estimates for the site-adjusted multivariable logistic regression model of factors associated with high-risk HPV (hrHPV) infection. (XLSX)

## References

[1] Okunade KS. Human papillomavirus and cervical cancer. J Obstet Gynaecol. 2019;40:602. doi: 10.1080/01443615.2019.1634030 PMC7062568 31500479

[2] Stelzle D, Tanaka LF, Lee KK, Ibrahim Khalil A, Baussano I, Shah ASV, et al. Estimates of the global burden of cervical cancer associated with HIV. Lancet Glob Health. 2021;9(2):e161–9. doi: 10.1016/S2214-109X(20)30459-9 33212031 PMC7815633

[3] Dryden-Peterson S, Bvochora-Nsingo M, Suneja G, Efstathiou JA, Grover S, Chiyapo S, et al. HIV infection and survival among women with cervical cancer. J Clin Oncol. 2016;34(31):3749–57. doi: 10.1200/JCO.2016.67.9613 27573661 PMC5477924

[4] AIDSinfo | UNAIDS DATA. 2025. [cited 2026 Jul 16]. Available from: https://aidsinfo.unaids.org/databook

[5] Bray F, Laversanne M, Hyuna S, Ferlay J, Siegel RL, et al. Global cancer statistics 2022: GLOBOCAN estimates of incidence and mortality worldwide for 36 cancers in 185 countries. CA Cancer J Clin. 2024;74:229–63. doi: 10.3322/CAAC.21834 38572751

[6] Wolf J, Kist LF, Pereira SB, Quessada MA, Petek H, Pille A, et al. Human papillomavirus infection: Epidemiology, biology, host interactions, cancer development, prevention, and therapeutics. Rev Med Virol. 2024;34(3):e2537. doi: 10.1002/rmv.2537 38666757

[7] World Health Organisation (WHO). IARC Monographs on the Evaluation of Carcinogenic Risks to Humans Human Papillomaviruses. 2007 [cited 2026 Jul 16]. Available from: https://publications.iarc.who.int/Book-And-Report-Series/Iarc-Monographs-On-The-Identification-Of-Carcinogenic-Hazards-To-Humans/Human-Papillomaviruses-2007

[8] World Health Organization. Cervical cancer. Available from: https://www.who.int/news-room/fact-sheets/detail/cervical-cancer

[9] World Health Organization. A cervical cancer-free future: First-ever global commitment to eliminate a cancer. 2020 [cited 2023 Sep 8]. Available from: https://www.who.int/news/item/17-11-2020-a-cervical-cancer-free-future-first-ever-global-commitment-to-eliminate-a-cancer

[10] Harper DM, Vierthaler SL. Next generation cancer protection: The bivalent HPV vaccine for females. ISRN Obstet Gynecol. 2011;2011:457204. doi: 10.5402/2011/457204 22111017 PMC3216348

[11] Joura EA, Giuliano AR, Iversen O-E, Bouchard C, Mao C, Mehlsen J, et al. A 9-valent HPV vaccine against infection and intraepithelial neoplasia in women. N Engl J Med. 2015;372(8):711–23. doi: 10.1056/NEJMoa1405044 25693011

[12] Markowitz LE, Tsu V, Deeks SL, Cubie H, Wang SA, Vicari AS, et al. Human papillomavirus vaccine introduction--the first five years. Vaccine. 2012;30 Suppl 5:F139–48. doi: 10.1016/j.vaccine.2012.05.039 23199957

[13] World Health Organization. Nigeria to vaccinate 7.7 million girls against leading cause of cervical cancer. 2023 [cited 2025 Sep 19]. Available from: https://www.afro.who.int/countries/nigeria/news/nigeria-vaccinate-77-million-girls-against-leading-cause-cervical-cancer

[14] Okoye JO, Ofodile CA, Adeleke OK, Obioma O. Prevalence of high-risk HPV genotypes in sub-Saharan Africa according to HIV status: A 20-year systematic review. Epidemiol Health. 2021;43:e2021039. doi: 10.4178/epih.e2021039 34044477 PMC8510839

[15] Mboumba Bouassa R-S, Gubavu C, Veyer D, Robin L, Gravier A, Hocqueloux L, et al. High prevalence of cervical high-risk human papillomavirus harboring atypical genotypes in human immunodeficiency virus -infected and -uninfected first-generation adult immigrant women originating from Sub-Saharan Africa and living in France. J Immigr Minor Health. 2021;23(2):308–19. doi: 10.1007/s10903-020-01074-7 32816173 PMC7914190

[16] Seyoum A, Assefa N, Gure T, Seyoum B, Mulu A, Mihret A. Prevalence and genotype distribution of high-risk human papillomavirus infection among Sub-Saharan African women: A systematic review and meta-analysis. Front Public Health. 2022;10:890880. doi: 10.3389/fpubh.2022.890880 35875040 PMC9304908

[17] Ezechi OC, Nwaokorie FO, Achaya I, Ujah O, Pettersson KO. The burden, distribution and risk factors for cervical oncogenic human papilloma virus infection in HIV positive Nigerian women. Virology J. 2014;11(1):5. 10.1186/1743-422X-11-5 PMC3896716 24433568

[18] Ezechi O, Akinsolu F, Salako A, Abodunrin O, Adewole I, Olagunju M, et al. High-risk human papillomavirus infection among Nigerian women: A systematic review and meta-analysis. J Int Med Res. 2023;51(7). doi: 10.1177/03000605231182884 37409466 PMC10331206

[19] Dom-Chima N, Ajang YA, Dom-Chima CI, Biswas-Fiss E, Aminu M, Biswas SB. Human papillomavirus spectrum of HPV-infected women in Nigeria: An analysis by next-generation sequencing and type-specific PCR. Virol J. 2023;20(1):144. doi: 10.1186/s12985-023-02106-y 37434253 PMC10337082

[20] Okwuraiwe AP, Ogbonne EL, Adeniyi AO, Ihurhe PI, Musa BO, Abe TR, et al. Detection of high-risk human papillomavirus genotypes among hiv-infected women in four states in Nigeria. Cureus. 2024;16(3):e57120. doi: 10.7759/cureus.57120 38681319 PMC11055541

[21] Olawepo JO, Ezeanolue EE, Ekenna A, Ogunsola OO, Itanyi IU, Jedy-Agba E, et al. Building a national framework for multicentre research and clinical trials: experience from the Nigeria Implementation Science Alliance. BMJ Glob Health. 2022;7(4):e008241. doi: 10.1136/bmjgh-2021-008241 35450861 PMC9024272

[22] Olakunde BO, Itanyi IU, Olawepo JO, Liu L, Bembir C, Idemili-Aronu N, et al. Comparative effectiveness of implementation strategies for Accelerating Cervical Cancer Elimination through the integration of Screen-and-treat Services (ACCESS study): Protocol for a cluster randomized hybrid type III trial in Nigeria. Implement Sci. 2024;19(1):25. doi: 10.1186/s13012-024-01349-9 38468266 PMC10926605

[23] Shi R, Qi W, Wang Z, Cai J, Zhao M, Wang Z. High-risk human papillomavirus genotype distribution and attribution to cervical lesions in a Shanxi Province screening population. Sci Rep. 2025;15(1):28217. doi: 10.1038/s41598-025-14228-0 40753301 PMC12317979

[24] Abi-Raad R, Sun T, Krishnamurti U. Human papillomavirus genotype distribution and its correlation with intraepithelial neoplasia, vaccination, and ethnicity. J Am Soc Cytopathol. 2025;14(4):273–8. doi: 10.1016/j.jasc.2025.03.006 40307088

[25] Murenzi G, Vuhahula E, Kimambo A, Matiku S, Tuyishime O, Liwa E, et al. High-risk human papillomavirus genotyping in cervical cancers in Tanzania. Infect Agent Cancer. 2024;19(1):35. doi: 10.1186/s13027-024-00596-1 39103953 PMC11301851

[26] Sweet K, Bosire C, Sanusi B, Sherrod CJ, Kwatampora J, Waweru W, et al. Prevalence, incidence, and distribution of human papillomavirus types in female sex workers in Kenya. Int J STD AIDS. 2020;31(2):109–18. doi: 10.1177/0956462419884454 31948341 PMC7031817

[27] Nakigozi H, Ndejjo R, Bazeyo W, Nabaggala A, Achola C, Iga M, et al. Prevalence of genital high-risk human papillomavirus infections and associated factors among women living with human immunodeficiency virus in Uganda. BMC Cancer. 2024;24(1):243. doi: 10.1186/s12885-024-11928-0 38383366 PMC10882885

[28] Kangethe JM, Gichuhi S, Odari E, Pintye J, Mutai K, Abdullahi L, et al. Confronting the human papillomavirus-HIV intersection: Cervical cytology implications for Kenyan women living with HIV. South Afr J HIV Med. 2023;24(1):1508. doi: 10.4102/sajhivmed.v24i1.1508 37928501 PMC10623654

[29] Bogale AL, Teklehaymanot T, Ali JH, Kassie GM, Medhin G, Baye AY, et al. Comparison of self-collected versus clinician collected cervicovaginal specimens for detection of high risk human papillomavirus among HIV infected women in Ethiopia. BMC Womens Health. 2022;22(1):360. doi: 10.1186/s12905-022-01944-2 36050660 PMC9434869

[30] Amare Y, Gelgalo D, Pozsgai É, Kiss I. Systematic review and meta-analysis of human papillomavirus prevalence and genotypic disparities among human immunodeficiency virus-positive women in Africa. J Clin Med. 2025;14(17):5924. doi: 10.3390/jcm14175924 40943685 PMC12429380

[31] Siqueira JD, Alves BM, Castelo Branco ABC, Duque KCD, Bustamante-Teixeira MT, Soares EA, et al. Comparison of four different human papillomavirus genotyping methods in cervical samples: Addressing method-specific advantages and limitations. Heliyon. 2024;10(3):e25474. doi: 10.1016/j.heliyon.2024.e25474 38327440 PMC10847660

[32] Bogale AL, Belay NB, Medhin G, Ali JH. Molecular epidemiology of human papillomavirus among HIV infected women in developing countries: Systematic review and meta-analysis. Virol J. 2020;17(1):179. doi: 10.1186/s12985-020-01448-1 33198743 PMC7670609

[33] Dastjerd NT, Abbasian L, Mohebbi SR, Banaem LM, Ashtiani MF, Rostamafshar Z, et al. Prevalence and distribution of human papillomavirus genotypes in women living with HIV: A cross-sectional study in Iran. BMC Infect Dis. 2025;25(1):1471. doi: 10.1186/s12879-025-11805-6 41184775 PMC12581437

[34] Okunade KS, Badmos KB, Okoro A, Awolola NA, Nwaokorie FO, Adelabu H, et al. Prevalence and associated factors of high-risk human papillomavirus infections among human immunodeficiency virus-infected women in Lagos, Nigeria. Res Sq. 2024. doi: 10.21203/rs.3.rs-4645178/v1 39070626 PMC11275992

[35] Sebsibe S, Molla G, Getachew B, Eshetu Y, Gedefie A, Erkihun Y, et al. Molecular epidemiology of high-risk human papillomavirus infection and its predictive factors among women living with HIV in Northeast Ethiopia: A facility-based cross-sectional study. BMC Infect Dis. 2025;25(1):1437. doi: 10.1186/s12879-025-11853-y 41162881 PMC12570764

[36] De Vuyst H, Steyaert S, Van Renterghem L, Claeys P, Muchiri L, Sitati S, et al. Distribution of human papillomavirus in a family planning population in Nairobi, Kenya. Sex Transm Dis. 2003;30(2):137–42. doi: 10.1097/00007435-200302000-00009 12567172

[37] Blossom DB, Beigi RH, Farrell JJ, Mackay W, Qadadri B, Brown DR, et al. Human papillomavirus genotypes associated with cervical cytologic abnormalities and HIV infection in Ugandan women. J Med Virol. 2007;79(6):758–65. doi: 10.1002/jmv.20817 17457908 PMC2846524

[38] Tounkara FK, Téguété I, Guédou FA, Goma-Matsétsé E, Koné A, Béhanzin L, et al. Human papillomavirus genotype distribution and factors associated among female sex workers in West Africa. PLoS One. 2020;15(11):e0242711. doi: 10.1371/journal.pone.0242711 33237976 PMC7688172

[39] Chachage M, Parikh AP, Mahenge A, Bahemana E, Mnkai J, Mbuya W, et al. High-risk human papillomavirus genotype distribution among women living with and at risk for HIV in Africa. AIDS. 2023;37(4):625–35. doi: 10.1097/QAD.0000000000003437 36398743 PMC9994804

[40] Ashry M, Shawky S, Mounir Z, Fathy F, Elsayed H, Kamal W, et al. Prevalence and risk factors of human papilloma virus infection among women living with HIV, Egypt, a cross sectional study. BMC Public Health. 2024;24(1):1821. doi: 10.1186/s12889-024-19240-z 38978047 PMC11232173

[41] Ermel A, Tong Y, Tonui P, Orang’o O, Muthoka K, Wong N, et al. Longer duration of anti-retroviral therapy is associated with decreased risk of human papillomaviruses detection in Kenyan women living with HIV. Int J STD AIDS. 2021;32(13):1212–20. doi: 10.1177/09564624211030766 34233531 PMC8608697

[42] Castle PE, Einstein MH, Sahasrabuddhe VV. Cervical cancer prevention and control in women living with human immunodeficiency virus. CA Cancer J Clin. 2021;71(6):505–26. doi: 10.3322/caac.21696 34499351 PMC10054840

[43] Song D, Li H, Li H, Dai J. Effect of human papillomavirus infection on the immune system and its role in the course of cervical cancer. Oncol Lett. 2015;10(2):600–6. doi: 10.3892/ol.2015.3295 26622540 PMC4509451

